# Calcium-Deficit Diet Improves Iron Content in Ovariectomized Rats

**DOI:** 10.1007/s12011-023-03556-9

**Published:** 2023-01-10

**Authors:** Joanna Suliburska, Natalia Wawrzyniak, Anna Gramza-Michałowska, Paweł Kurzawa

**Affiliations:** 1 Department of Human Nutrition and Dietetics, Faculty of Food Science and Nutrition, Poznań University of Life Sciences, Wojska Polskiego 31, 60-624 Poznań, Poland; 2 Department of Gastronomy Science and Functional Foods, Faculty of Food Science and Nutrition, Poznań University of Life Sciences, Wojska Polskiego 31, 60-624 Poznań, Poland; 3 Department of Clinical Pathology, Poznań University of Medical Sciences, Przybyszewskiego 49, 60-355 Poznań, Poland; 4 Department of Oncological Pathology, University Hospital of Lord’s Transfiguration, Partner of Karol Marcinkowski University of Medical Sciences, Szamarzewskiego 84, 60-569 Poznań, Poland

**Keywords:** Ovariectomy, Iron, Calcium, Rat

## Abstract

In women, menopause is associated with disorders related to calcium and iron content, which may increase the risk of osteoporosis. This study aimed to determine the effect of calcium deficiency on the iron content in ovariectomized rats. This study included 30 3-month-old female rats, which were divided into three groups: group C (*n* = 10)—control group fed the standard diet; group O—ovariectomized rats fed the standard diet; and group D—ovariectomized rats fed the calcium-deficit diet. After 3 months of experimental intervention, the weight of the rats was measured, and blood and tissue samples were collected. Morphological parameters were analyzed in whole blood, and serum levels of leptin, estrogen and C-reactive protein, and total antioxidant status were determined. The iron content was measured in tissues, and histological analysis was performed in the femur. The results obtained demonstrated that ovariectomy significantly decreased the iron content in bones, hair, spleen, liver, and kidneys. The calcium-deficit diet increased the iron content in tissues and the hemoglobin level in ovariectomized rats and also enhanced the number of osteoblasts in bones compared with the O group. In conclusion, calcium deficiency improved the iron content in ovariectomized rats in this 12-week study.

## Introduction

Menopause and decreased estrogen levels increase the risk of osteoporosis in women [1]. An important factor driving unfavorable changes in bone structure is inappropriate nutrition, especially low intake of calcium and vitamin D [2–4]. In postmenopausal women, calcium deficiency and disorders of calcium metabolism coexist with iron accumulation in the body which is mainly indicated by the high concentration of ferritin [5]. In postmenopausal women, due to the cessation of menstruation, iron loss via the menstrual blood does not occur, which explains the increase in the iron content [6]. An excessive iron content is associated with the acceleration of osteoporotic changes. It is associated with the accumulation of iron in bones, the increase in oxidative stress, and the disturbance of osteoblast differentiation [7]. Thus, an inappropriate supply of calcium and iron may contribute to osteoporotic changes in bones. The interaction between calcium and iron during their absorption in the small intestine is well known [8–10]. Many clinical and experimental studies have reported that calcium supplementation reduces the bioavailability of iron with no impact on the hemoglobin level [11]. In addition, some studies have shown that an increased supply of iron salts affects the calcium content in the body [12, 13]. Many studies have investigated the use of calcium supplements in menopausal women, including their effects on iron metabolism. However, no study has explored how dietary calcium deficiency under menopausal conditions and low estrogen levels (a common relationship observed in menopausal women) affects the iron content in the body. Therefore, in this study, the effect of ovariectomy with normal and deficient dietary calcium levels on the iron content was investigated in rats.

## Material and Methods

### Study Design

This study was approved by the Local Ethics Committee in Poznan, Poland (registration number: 34/2019). Thirty female Wistar rats aged 3 months were included in this study. The rats were obtained from the Greater Poland Center for Advanced Technologies, University of Adam Mickiewicz in Poznan, Poland.

The rats were adapted to laboratory conditions over the first 7 days. Twenty rats were subjected to ovariectomy. After the surgery, the rats were fed a semisynthetic diet based on the AIN-93 M diet [14] and distilled water. The control group (C group) constituted ten rats fed the AIN-93 M diet and distilled water ad libitum. After 7 days of recovery, the ovariectomized rats were divided into two groups: those fed the standard diet (O group, *n* = 10) and those fed the calcium-deficient diet (D group, *n* = 10). In the latter, calcium was not added to the mineral mixture of AIN-93 M. The detailed experimental protocol and dietary components were presented in our previous studies [15].

After 12 weeks of the experiment, all rats were euthanized by decapitation (after 12 h of fasting). Blood samples were collected to obtain whole blood for morphological analysis, and a part of the blood was stored in serum-separated tubes to obtain serum. The coagulated blood was centrifuged at 20,000 rpm for 15 min at 4 °C. The serum was separated and stored in Eppendorf tubes at − 80 °C. The liver, kidneys, heart, spleen, pancreas, brain, hair, and femur were dissected, washed using saline, weighed, and stored at − 80 °C. The right femur was frozen, and the left femur was immersed in formalin. Hair was collected from the interscapular area. All rats were fed diets and distilled water ad libitum. The intake of the diet was monitored daily, and body weight was measured weekly.

### Fe Analysis in Diets

To determine the iron content in the diets, 1 g of each sample of the diet was mineralized in a muffle furnace at 450 °C and then dissolved in 1 N nitric acid (Merck, Kenilworth, NJ, USA). The iron content was determined using flame atomic absorption spectrometry (AAS-3, Carl Zeiss, Jena, Germany) after diluting the samples with deionized water. This method was validated with an accuracy of 95% using brown bread (BCR191, Sigma-Aldrich, St. Louis, MO, USA)—a certified reference material. All diet samples were analyzed in triplicate.

### Fe Analysis in Tissues

To determine the iron content in tissues, the samples were mineralized in a microwave digestion system (Speedwave Xpert, Berghof, Eningen, Germany) using pure nitric acid (Merck, Kenilworth, NJ, USA), and then the iron content was determined using flame atomic absorption spectrometry (AAS-3, Carl Zeiss, Jena, Germany). This method was validated with an accuracy of 97% using bovine liver (1577C, Sigma-Aldrich, St. Louis, MO, USA)—a certified reference material.

### Biochemical and Morphological Measurements

Whole-blood morphological analysis was carried out on the day of blood collection in a commercial laboratory (Alab, Poznań, Poland). Serum levels of leptin, estrogen and C-reactive protein, and total antioxidant status (TAS) were determined using ELISA kits (SunRed, Shanghai, China). These kits were based on the principle of the dual antibody sandwich technique for the detection of parameters in rats. The analysis was carried out using an infinite F50 spectrometer (Tecan Group Ltd., Männedorf, Switzerland). Reproducibility was confirmed using the control serum sample provided by the kit manufacturer.

### Histological Analysis

The resected femoral bones were fixed with 10% buffered formalin for 24 h and then decalcified in the Osteodec bone marrow biopsy decalcifying solution for further 3 h. Subsequently, each specimen was routinely processed and embedded separately in paraffin blocks. From each block, three 2-µm-thick sections were cut and stained with hematoxylin and eosin. Each slide contained two femoral bone sections with the bone marrow content. The bone marrow of each bone was analyzed using a light microscope (Leica, Allendale, NJ, USA). The number of osteoblasts and osteocytes was counted in each HPF area.

### Statistical Analysis

Data were expressed as means ± standard deviation. The Shapiro–Wilk test was used to verify the normality of the distribution of the variables. Statistical significance was determined using a one-way analysis of variance with Tukey’s test, using the Statistica software (StatSoft, Tulsa, USA). The difference was considered significant when *p* < 0.05.

## Results

**Fig. 1:**
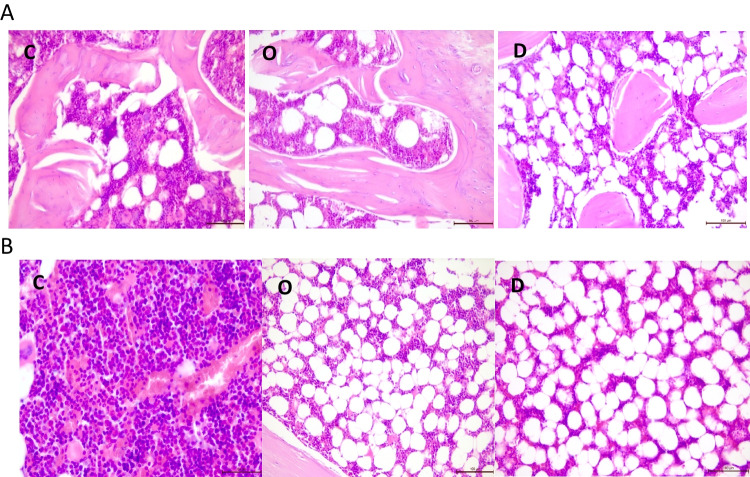
**A** number of osteoblasts in femur (H&E; 200 ×). **B** Marrow cellularity (H&E: C, 400 × ; O, D 200 ×)

Body mass, selected biochemical and morphological parameters, iron content in tissues, and histological analysis parameters of femurs are presented in Tables 1, 2, and 3. Ovariectomy significantly increased the body mass and the serum leptin level and decreased the serum estrogen level. Compared with the C group, the hemoglobin level in whole blood was markedly higher, and the percentage of red blood cell distribution width and the coefficient of variation (RDW-CV) in the blood were markedly lower in the D group. A significantly lower iron content in bones, hair, spleen, liver, and kidneys was observed in the O group than in the C group. Compared with the O group, the iron content was markedly increased in bones, spleen, liver, and kidneys in the D group. Marrow cellularity decreased dramatically after ovariectomy (in O and D groups), and the number of osteoblasts was significantly higher in the D group compared with C and O groups. Moreover, significantly positive correlations were observed between the iron content in bones and marrow cellularity (*r* = 0.69) and TAS (*r* = 0.42). A positive relationship was also observed between red blood cells and erythroid lineage (*r* = 0.43), whereas a negative correlation was observed between the iron content in bones and the number of osteocytes (*r* =  − 0.39). The number of osteoblasts and marrow cellularity in the femur of rats in three groups is presented in Fig. 1.

**Table 1:** Body mass, daily intake of diet, and iron in rats

Parameter	Group		
	C	O	D
Body mass (g)	325.86 ± 25.97^a^	421.90 ± 55.10^b^	441.90 ± 70.97^b^
Daily diet intake (g)	25.08 ± 0.63	25.11 ± 1.70	26.14 ± 1.87
Daily Ca intake (mg)	141.12 ± 3.56^b^	141.30 ± 9.57^b^	16.77 ± 1.20^a^
Daily Fe intake (mg)	0.90 ± 0.02	0.90 ± 0.06	0.93 ± 0.07

*C*, control group; *O*, ovariectomy group with standard diet; *D*, ovariectomy group with calcium deficit diet

^a,b^Significant differences between groups (*p* < 0.05)

**Table 2:** Blood and tissue parameters in rats

Parameter	Group		
	C	O	D
Biochemical parameters			
Leptin (ng/ml)	0.98 ± 0.50^a^	2.69 ± 1.36^b^	2.39 ± 1.42^b^
ES (ng/l)	49.82 ± 4.62^b^	21.68 ± 6.8^a^	19.46 ± 4.22^a^
CRP (pg/ml)	814.87 ± 169.49	784.86 ± 220.72	702.65 ± 112.49
TAS (U/ml)	10.25 ± 2.83	8.69 ± 1.77	9.24 ± 1.64
RBC (T/l)	8.30 ± 0.38	8.45 ± 0.26	8.53 ± 0.28
HB (g/dl)	15.30 ± 0.69^a^	15.77 ± 0.65^ab^	16.12 ± 0.48^b^
HCT (%)	43.00 ± 1.52	43.26 ± 1.64	44.52 ± 1.62
MCV (fl)	51.87 ± 1.81	51.23 ± 1.87	52.24 ± 1.75
MCH (pg)	18.58 ± 0.52	18.63 ± 0.87	18.93 ± 0.52
MCHC (d/dl)	35.86 ± 0.84	36.38 ± 0.78	36.23 ± 0.80
PLT (G/l)	792.10 ± 337.74	847.00 ± 216.32	861.50 ± 288.38
RDW-CV (%)	14.88 ± 1.07^b^	14.05 ± 0.88^ab^	13.13 ± 0.56^a^
Fe concentration in tissues (µg/g dry mass)			
Bone	88.20 ± 8.48^c^	55.93 ± 9.24^a^	68.26 ± 12.95^b^
Pancreas	110.98 ± 11.19	101.17 ± 11.76	106.68 ± 4.54
Hair	176.85 ± 15.38^b^	126.15 ± 8.02^a^	132.24 ± 7.25^a^
Spleen	10,052.40 ± 2090.40^c^	5111.12 ± 842.59^a^	7314.43 ± 2018.32^b^
Liver	1698.31b ± 221.86^b^	1096.09 ± 168.40^a^	1526.36 ± 152.71^b^
Heart	516.51 ± 35.26	540.38 ± 37.98	537.00 ± 38.38
Brain	131.13 ± 17.81	144.08 ± 19.40	132.00 ± 20.58
Kidney	790.27 ± 87.97^b^	589.47 ± 64.66^a^	747.17 ± 91.03^b^
Bone parameters			
Number of osteoblasts	9.90 ± 3.41^a^	9.00 ± 5.26^a^	15.13 ± 3.55^b^
Number of osteocytes	38.40 ± 7.47	45.00 ± 8.46	40.50 ± 7.68
Marrow cellularity (%)	91.5 ± 3.07^b^	57.00 ± 10.91^a^	58.00 ± 8.33^a^
Erythroid lineage (%)	37.00 ± 4.83	36.00 ± 5.00	36.00 ± 6.97

*C*, control group; *O*, ovariectomy group with standard diet; *D*, ovariectomy group with calcium deficit diet, *ES*, estrogen; *CRP*, C-reactive protein; *TAS*, total antioxidant status; *RBC*, red blood cells; *HB*, haemoglobin; *HCT*, haematocrit; *MCV*, mean corpuscular volume; *MCH*, mean corpuscular haemoglobin; *MCHC*, mean corpuscular haemoglobin concentration; *PLT*, platelets; *RDW-CV*, red blood cell distribution width, coefficient of variation

^a,b,c^Significant differences between groups (*p* < 0.05)

**Table 3:** Significant correlation between parameters

Parameters	***r***
Fe_bone (µg/g dm) and marrow cellularity (%)	0.69
Fe_bone (µg/g dm) and number of osteocytes	− 0.39
RBC (T/l) and erythroid lineage	0.43
Fe_bone (µg/g dm) and TAS (U/ml)	0.42

*dm*, dry mass; *RBC*, red blood cells; *TAS*, total antioxidant status; *r*, correlation coefficient

## Discussion

The most important findings of this study are that ovariectomy decreased the iron content in tissues and that calcium deficiency improved the iron content and increased the hemoglobin level in ovariectomized rats.

In this study, the serum estrogen level decreased significantly along with an increase in the body mass (and the serum leptin level) and a decrease in the iron content in tissues after ovariectomy. However, calcium deficiency increased the iron content with no changes in the body mass and the estrogen level compared with the O group. These results differ from the observations in postmenopausal women, in whom a decrease in the estrogen level is reported to cause osteoporosis, which is exacerbated by the excessive iron content in the body [16, 17]. The findings of the present study are consistent with those of other animal studies, in which no iron accumulation occurred in ovariectomized rats [18]. The poor iron content in ovariectomized rats may be related to the low estrogen level. Previous studies have reported that the relationship between the estrogen level and iron absorption and transport may be due to the regulating effect of the estrogen level on iron regulatory proteins in the organism [18]. Moreover, another study has reported that estrogen treatment in ovariectomized rats may inhibit the iron declining effect [19]. Ikeda et al. have reported that estrogen regulates hepcidin expression via gpr30-bmp6-dependent signaling in hepatocytes [20]. This mechanism may also relate the estrogen level to iron metabolism. However, in the study of Ikeda et al., an increased iron content in the serum and liver and upregulation of ferroportin in the duodenum were observed in ovariectomized mice. Several studies have reported that iron content disorders affect bone metabolism and significantly decrease bone mass density (BMD) due to oxidative stress in postmenopausal women and ovariectomized rats or mice [21]. In addition, reactive oxygen species may act as a mediator in RANKL-induced signaling pathways by enhancing the differentiation of osteoclasts and, consequently, bone loss [22]. Moreover, ferritin—an iron storage protein—is found to inhibit osteoblast activity [23]. In the present study, the number of osteoblasts and the iron content in the femur were increased in the D group; however, the iron content in bones correlated negatively with the number of osteocytes. This may confirm the inhibition effect of the increasing iron content in bones on the differentiation of osteoblasts into osteocytes. In this study, ovariectomy decreased bone marrow cellularity, which was positively correlated with the iron content in bones. Low marrow cellularity after ovariectomy is related to the increase in the adipocyte content in the bone marrow, as reported in our previous work [24]. Moreover, the correlation between the iron content in bones and TAS is probably attributable to changes caused by ovariectomy, i.e., a decrease in the iron content and an increase in oxidative stress. Similar changes in Fe and TAS were observed in individual groups although these changes were not significant for TAS.

Calcium deficiency and osteoporosis are common in postmenopausal women, and the same was observed in the D group. Interestingly, the low calcium content improved the iron content in tissues in rats. This finding seems to be beneficial in terms of the iron content, but cannot be generalized for postmenopausal conditions. Hence, postmenopausal women may exhibit iron accumulation; furthermore, low estrogen levels and iron overload may increase bone loss, and calcium deficit may enhance these changes [25]. This is consistent with the findings of the present study because in the present study, calcium deficiency and low estrogen levels increased the iron content in many tissues and also bones. Dietary calcium intake affects the iron content due to calcium–iron interactions at the absorption level, and the effects of this interaction are particularly evident in a deficiency state [9, 26, 27]. Some studies have indicated that calcium supplementation decreases iron absorption and that a high iron supply inhibits calcium bioavailability. The mechanism responsible for these processes may involve the activity of divalent metal transporter 1 in the duodenum and upregulation of duodenal claudin-2, a cation-selective pore-forming protein [28]. Improvement in the iron content in calcium deficiency, along with a higher hemoglobin level and a lower RDW-CV percentage in the blood, was observed in this study. In some clinical studies, osteoporosis was associated with a low hemoglobin level, but higher levels of hemoglobin were also found to play a protective role against osteoporosis [29]. Recently, studies on human participants have reported a nonlinear relationship between the hemoglobin level and lumbar and thoracic BMD, and this relationship is dependent on age and, probably, the estrogen level [30]. Chuang et al. [31] have reported a negative correlation between the hemoglobin level and BMD in women aged below 50 years but a positive correlation between the same parameters in women aged above 50 years. In the present study, the high hemoglobin level in the D group was probably due to the increased iron content.

This study has some limitations. The experiment lasted 12 weeks, and this may have affected the results obtained. Only selected parameters of the iron content and bone histological analysis were analyzed. Calcium parameters were not included, and the vitamin D level was not analyzed. Moreover, a sham-operated group was not included, but ovariectomized rats were compared with the control group.

## Conclusion

Based on the results obtained, it can be concluded that the calcium-deficit diet improves the iron content in ovariectomized rats and that the iron content in bones is associated with bone structure parameters and TAS.

In order to confirm the obtained results, further long-term studies in rats in the model of postmenopausal osteoporosis are required. The results of this study show that the study of postmenopausal osteoporosis should take into account the supply of calcium and iron and their mutual relationships in the body, which may affect the development of osteoporosis. In the future, the obtained results will help to understand the mechanism of osteoporosis development in postmenopausal women and determine the factors of prevention and/or treatment of this disorder.

## Data Availability

The datasets used and/or analyzed in the current study are available from the corresponding author upon reasonable request.
